# Rearing Honey Bees, *Apis mellifera, in vitro* 1: Effects of Sugar Concentrations on Survival and Development

**DOI:** 10.1673/031.011.9601

**Published:** 2011-07-27

**Authors:** Osman Kaftanoglu, Timothy A. Linksvayer, Robert E. Page

**Affiliations:** ^1^ School of Life Sciences, Arizona State University, Tempe, AZ. 85287-4501, USA; ^2^ Present Address: Department of Biology, University of Pennsylvania, 225 Leidy Laboratories, 433 South University Avenue, Philadelphia PA 19104-6018

**Keywords:** larval development, live weights, ovarioles, carbohydrate

## Abstract

A new method for rearing honey bees, *Apis mellifera* L. (Hymenoptera: Apidae), *in vitro* was developed and the effects of sugar concentrations on survival and development were studied. Seven different glucose (G) and fructose (F) compositions (0%G+0%F, 3%G+3%F, 6%G+6%F, 12%G+12%F, 0%G+12%F, 12%G+0%F, and 4%G+8%F) were tested. Larvae were able to grow to the post defecation stage without addition of sugars (Diet 1), but they were not able to metamorphose and pupate. Adults were reared from diets 2–7. The average larval survival, prepupal larval weights, adult weights, and ovariole numbers were affected significantly due to the sugar compositions in the diets. High sugar concentrations (12%G+12%F) increased the number of queens and intercastes.

## Introduction

Honey bee larvae, *Apis mellifera* L. (Hymenoptera: Apidae), are fed mandibular and hypopharyngeal gland secretions produced by nurse bees. This food contains all the nutrients necessary for the development of queens, workers, and drones (Haydak 1970; Johansson 1955; Rembold 1965; Weaver 1966; Brouwers 1984; Howe et al. 1985).

A queen larva is fed a total of 1600 times, and these feeding events last a total of 17 hours. The number of feeding events increases with age from about 13 feedings/h at 1 day old, 16 feedings/h at 3 days, and 25 feedings/h at 4 days old (Jung-Hoffmann 1966; Haydak 1970). Queen-destined larvae receive abundant fresh royal jelly and grow at least 1500–1700 times of the weight of the egg from 0.12–0.20 mg (Taber and Roberts 1963; Roberts and Taber 1965) up to 250–346 mg (Haydak 1970; Winston 1987). Young worker-destined larvae up to 2.5 days old receive the same brood food as queen larvae, and like queen larvae they float on the food. However, after the third day they receive restricted food and finish all the food that is given to them. It is estimated that a worker larva is fed by the nurse bees 143 times lasting about 2 hours during the whole larval stage (Lindauer, 1952). Although the genotype, cell size, nutrition, and season influence the size and the weights of resulting adult worker bees, a worker larva grows about 900–1100 times the weight of an egg or newly hatched larva (Wang 1965).

Many attempts have been made to rear *A. mellifera* in the laboratory (Michael and Abromovitz 1955; Weaver 1955, 1958, 1962, 1970, and 1974; Smith 1959; Hoffmann 1960; Mitsui et al. 1964; Rembold 1965; Jay 1965; Rembold et al. 1974; Asencot and Lensky 1976; Schuel et al. 1978; Schuel and Dixon 1968, 1986), but the methods were labor intensive, yielded small numbers of viable individuals, and were not sufficient to consistently produce individuals of specific castes.

Rembold and Lackner (1981) developed a larval diet for rearing queens *in vitro* by using 20 g royal jelly, 2.5 g D-glucose, 2.5 g D-fructose, and 20 ml distilled water. They obtained 75 % adult survival and most adults were workers. Adding 0.5 g Difco bacto-yeast extract to this basic food increased the survival of the larvae to 80% and 30% of the individuals developed into queens.

Hanser (1983) used royal jelly diluted with nutrient solution which consisted of 35 g of honey, 10 g of Torula yeast, 0.3 g Nipagin, and 100 ml double-distilled water for *in vitro* rearing. For feeding younger larvae royal jelly was diluted at 2:1 ratio and for older larvae 1:1 ratios. One to two days old 4–5 larvae were grafted on 0.25 ml of royal jelly solution. They were kept at 35° C and 85–90% RH in an incubator and fed twice a day with 0.1 ml royal jelly solution. Unconsumed royal jelly was removed from the cups and fresh food was supplied. Just before they started spinning, they were transferred to new cups and pupated in the incubator.

Vandenberg and Shimanuki (1987) developed a technique to rear honey bee larvae in plastic and beeswax queen cell cups, fed them with the mixture of 50 % royal jelly, 6 % Dglucose, 6% D-fructose, 1 % yeast extract, and 37 % distilled water in an incubator which was kept at 34° C and 96% RH during larval stage and 70% RH during pupal stages. They obtained up to 90% larval survival in beeswax and 57 % survival in plastic cell cups. Peng et al. (1992) modified Vandenberg and Shimanuki's method and raised larvae in 24 well plates to study the effects of Chlortetracycline on the development of worker larvae reared *in vitro*. Their modified basic larval diet consisted of 4.2 g royal jelly powder, 0.6 g glucose, 0.6 g fructose, 0.2 g Difco yeast extract, and 14.4 g doubledistilled water. The larval mortality and the post-defecation mortality rates were 6.3% and 18.1%, respectively.

Aupinel et al. (2005) also used Vandenberg and Shimanuki's diet and improved the technique by altering the quantity (130 µl vs. 160 µl), sugar content (6%, 7.5%, 9%), and the yeast extract content (1%, 1.5%, 2%) of the diet at different instars. They used plastic queen cell cups and placed them in 48 well plates and fed them once a day. The larvae fed with 160 µl of diet were heavier than larvae fed with 130 µl diet. The survival rate also was increased from 33.64% to 69.7% when the amount of food increased. Brodshnider et al. (2009) reared bees *in vitro*, introduced them into field colonies and observed the flight behavior and compared it to natural workers. They did not find morphological difference between the hive reared bees and *in vitro* reared bees.

In all the above methods, fresh or freshly lyophilized royal jelly was used for the preparation of the larval diets. Larvae were either kept in groups of 10, 3, 2 or 1 in 24 well plates (Peng et al. 1992) or in individual queen cell cups (Vandenberg and Shimanuki 1987; Aupinel et al. 2005). We built on these previous approaches and a simple method was developed to raise worker *A. mellifera* using cheap commercially available royal jelly.

**Table 1.**
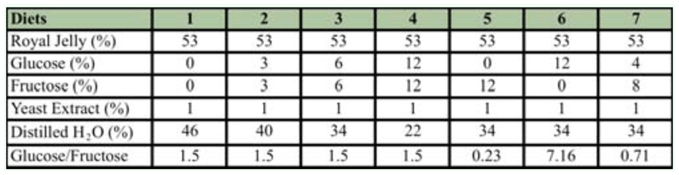
The composition of diets used to feed *Apis mellifera* larvae *in vitro*.

## Materials and Methods

Worker larvae were obtained from the honey bee (*Apis mellifera ligustica*) colonies at the Honey Bee Research Facility, School of Life Sciences, Arizona State University, Mesa, Arizona. Queens were confined to a fully drawn comb in an excluder cage (46 × 24 × 6 cm) as described by Peng et al. (1992). On the fifth day, the bees were shaken off the comb and the comb was brought into the grafting room to obtain 1.5–2 day old larvae.

Seven different larval diets were prepared by changing the sugar and water concentrations (Table 1). Sugars and the yeast extract were dissolved in distilled water and freshly thawed commercial royal jelly purchased from a local bee supply company was added to the mixture and mixed thoroughly on a shaker. The diets were divided into 2 ml centrifuge tubes and kept at -18° C in a freezer until they were used. The diets were thawed and brought to 34° C in a water bath just before feeding.

**Figure 1.**
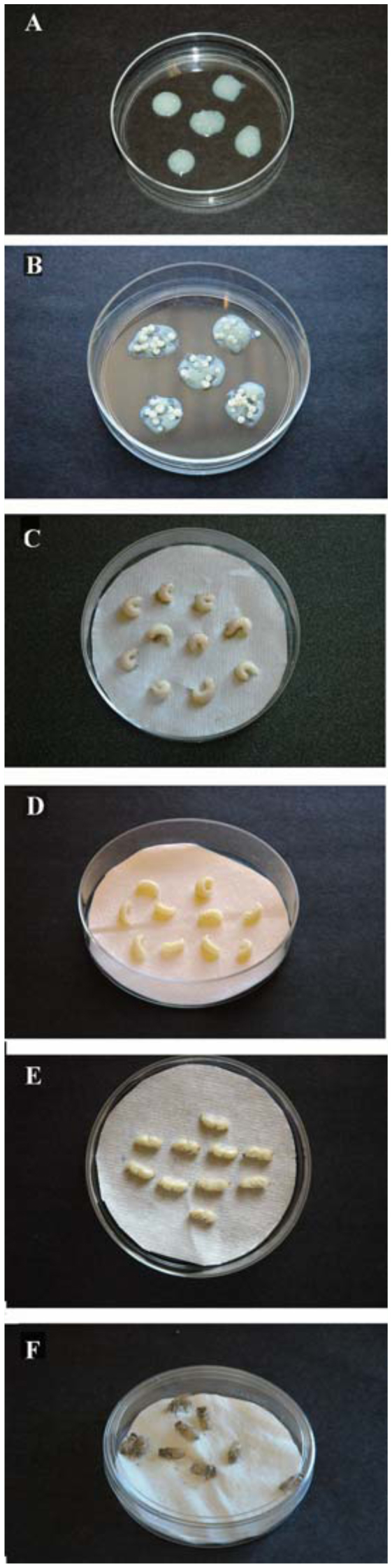
Rearing *Apis mellifera in vitro*. (A) Newly grafted larvae into aliquots of diets; (B) Three days after grafting; (C) Transferring larvae on filter paper; (D) Post defecation or spinning stage; (E) Early pupal stage; (F) Adult bees reared *in vitro*. High quality figures are available online.

A total of 350 larvae were grafted; there were 7 treatment groups, 5 replicates, and 10 larvae in each replicate. The first day 5 aliquots of 200 mg food were placed in a polyethylene Petri dish (100 × 15 mm) and 10 larvae were grafted on each aliquot (Figure 1A). The Petri dishes were placed into a polyethylene tub (20 cm × 40 cm) containing 16% sulfuric acid, transferred into a humidity chamber, and kept there at 34° C and 90% RH. On the second day, 40 mg and the 3^rd^ day 80 mg of larval food/larvae were placed in new Petri dishes and the larvae were gently placed on top of the fresh food (Figure 1B). On the 4^th^ day, 120 mg and on the 5th day 180 mg of food/larvae was placed in Petri dishes and the larvae were transferred onto the food. On the 6th day larvae consumed most of the food, and they started depositing uric acid crystals on the dorsal side of the body. When uric acid crystals were observed, the larvae were removed from the feeding dishes, weighed, and transferred to a 100 × 15 mm Petri dishes lined with Kimwipes® tissue paper (Figure 1C).

The next day the old Kimwipes® tissue paper containing feces was removed and the larvae were gently transferred onto a clean tissue paper and kept at 34° C and 70% RH in the humidity chamber. At the end of the defecation stage larvae started spinning cocoons, and this was recorded as the spinning stage (Figure 1D). Dead or undeveloped pupae were removed from the Petri dishes, and pupae (Figure 1E) were kept in the humidity chamber until they completed development and became adults (Figure 1F). Bees were removed from the Petri dishes as soon as they become adults, weighed, inspected under a stereo microscope, and dissected to count the ovarioles.

The adult bees were classified as queen phenotypes if they completed the development in 15–16 days, had notches on the mandibles, curved stings, large spermathecae (1mm in diameter), and had no corbiculae; as worker phenotypes if they completed the development in 21–22 days, had rows of corbicular hairs, straight stings with barbs, and had mandibles without the notches; and as intercastes if they completed development between 17–20 days, had small notches on the mandibles, and/or undeveloped corbiculae.

Hive-reared *A. mellifera* served as controls. The brood comb from which larvae were grafted was removed from the colony 20 days after caging the queen and placed in an incubator. The next day newly-emerged bees were sampled, weighed, and dissected for ovariole counts.

**Table 2.**
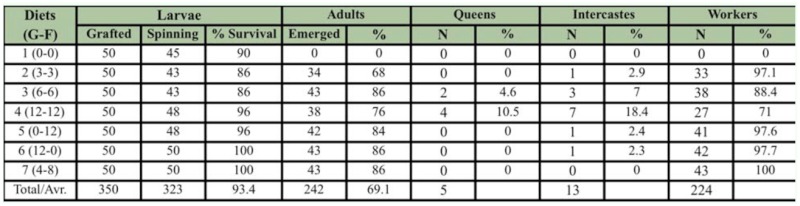
Effects of different sugar compositions on the survival and development of *Apis mellifera* larvae reared *in vitro*.

**Table 3.**
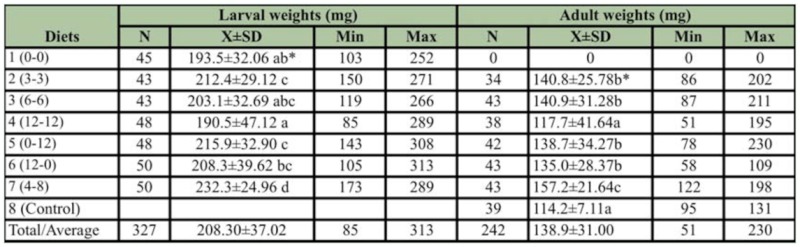
The effects of different sugar concentrations on larval weights (mg) before defecation and adult weights (mg) of *Apis mellifera.*

## Results

### Effects of sugar concentrations on the survival

Larval and adult survival rates are summarized in Table 2. The results showed that *A. mellifera* larvae can develop with a mixture of royal jelly, yeast extract, and water, without the addition of carbohydrates. However, they cannot pupate and become adults if there are not enough carbohydrates in the diet.

When the adult survival rate of diet 1 was excluded, the average survival rate increased from 69.1–81%. Development rates of larvae to the adult stage were lower in diets 1, 2, and 4 than the others. It was observed that the survival rates were higher in diets containing 12% sugar than 6 or 24%. The glucose and fructose ratio was not crucial for the survival of the developing larvae, and they were able to utilize both sugars effectively.

### Effects of sugar concentrations on predefecation larval weights, adult weights and ovariole numbers

The average larval weight was 208.30 ± 37.02 mg among the groups and the sugar composition of the food affected the larval weights significantly (ANOVA F_6,326_ = 8.027, *p* < 0.001); being the highest in the Diet 7 and lowest in the Diets 1 and 4 (Table 3).

Sugar composition of the diets also affected the live weights of the adult bees significantly. Larvae that were fed diet 7 had the highest adult weights (157.2 ± 21.64 mg) and they were heavier than the other groups (ANOVA F_6,_281 = 11.897, *p* < 0.001). Larvae that were fed diet 4 and the hive-reared controls had the lowest adult weights, and weighed 117.7 ± 41.64 mg and 114.2 ± 7.11 mg, respectively. The other groups were between the control group and the diet 7.

**Table 4.**
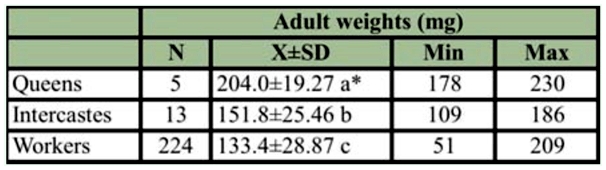
The average weights of the queen, intercastes and worker *Apis mellifera* reared *in vitro*.

**Table 5.**
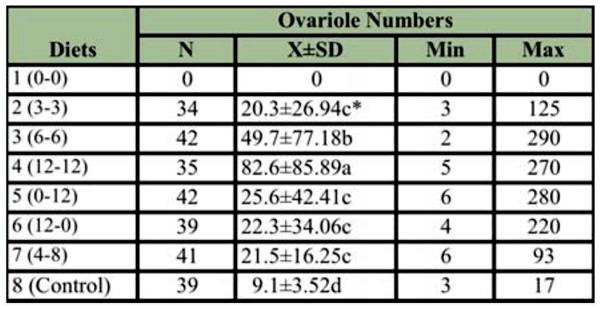
Average ovariole number of the *Apis mellifera* reared on different diets *in vitro*.

The average weight of the *in vitro* reared queens, intercastes, and worker adults were 204.0 ± 19.27 mg, 151.8 ± 25.46 mg, and 133.4 ± 28.87 mg, respectively (Table 4). The average weights (204.0 ± 19.27 mg) of *in vitro* reared queens were similar to that of hive—reared queens.

The ovariole numbers of the adults reared *in vitro* were also affected by the sugar composition of the diets significantly (ANOVA F_6_,271 = 9.378, *p* < 0.001). Adults that were reared on diet 4 had the highest number of ovariole numbers (Table 5). They received the highest sugar composition in the diet. Even though the larvae consumed less food, the high sugar composition in the diet increased the development of queens and intercastes. As a consequence they had significantly more ovarioles than the other groups. Larvae that were fed diet 3 (6% G and 6% F) had more ovarioles than low sugar diet (3% F), single sugar diets (diets 5 and 6), and fructose biased diet (diet 7). There was not a significant difference (*p* > 0.05) between ovariole numbers reared on single sugar diets (diet 5 and 6), low sugar diet (diet 2), or fructose biased diet (diet 7). Hive-reared control adults had the lowest ovariole numbers. Since they received restricted diet during larval stages their size and the ovariole numbers were regulated by the amount of food, composition of food, and the cell size.

## Discussion

*Apis mellifera* larvae can survive and grow on an artificial diet composed of royal jelly, sugars, yeast extract, and distilled water. Glucose and fructose composition of the basic larval diet did not affect the survival rates of the larvae reared *in vitro*. However, the sugar composition significantly affected the development of adults (0% in diet 1, to 86% in diets 3, 6, and 7).

Sugar composition of the diets also affected the pre-defecation larval weights. The weight of the larvae reared with diet 7 was significantly higher than the others (*p* < 0.001) and they all developed into workers. Brouwers (1984) showed that sugar composition was 20% in royal jelly and 15% in worker jelly collected from the cells containing 1–3 days old larvae. Glucose to fructose ratio in worker jelly was 1.3 at younger ages and it decreased to 0.7 at older ages, whereas these ratios in royal jelly were 1.7 at younger ages and decreased gradually, but remained higher than 1, at older ages. Glucose was predominant during the early larval stages of workers, but fructose became the main sugar component in the food of older larvae. (Brouwers 1984; Beetsma 1985; Brouwers et al. 1987). Diet 7 resembles the worker jelly in terms of glucose/fructose ratio and resulted in the development of workers, rather than the development of intercastes or queens.

Asencot and Lensky (1976) reported that larvae reared on worker jelly only were unable to pupate and metamorphose. Pupation and emergence of adult workers were achieved by supplementing worker jelly with glucose and fructose. Addition of 200 mg (20%) of glucose and 200 mg of fructose to the diet increased the survival rate to 76.7% and development of 50% queens, 40.9% intercastes, and 9.1% workers. In our experiments high sugar concentration also increased the development of queens and intercastes as reported by Asencot and Lensky (1976). The high sugar diet (24% in diet 4) yielded 4 fully developed queens, 7 intercastes, and 27 worker bees. Diet 3 (12% sugar) also yielded 2 fully developed queens and 3 intercastes. Diets 2, 5, and 6 yielded only one intercaste, but no queens. Only workers developed from diet 7.

In general, larval weights change by the amount of food, and the moisture content of the food supplied to the larvae. Dietz and Haydak (1971) were able to raise more queens by increasing the moisture content of the food. It has also been reported that the addition of water to lower the total solids of royal jelly improved larval growth (Haydak 1943; Smith 1959; Weaver 1955). We observed the same phenomenon. Larval weights were significantly lower in diet 4, even though it had more sugars than the other diets. This diet had less water content and was thicker. Therefore, the larvae did not consume as much food in this diet, and this resulted in development of smaller larvae.

Even though the moisture content was the highest in diet 1, larvae were not able to pupate without the addition of sugars to the diet. Similar results were reported by Asencot and Lensky (1976).

Diet 4 produced a higher proportion of queens and intercastes; therefore, it is not recommended for rearing worker larvae *in vitro*. Even though the adults reared on diet 7 were heavier than hive-reared adults, it yielded 100 % worker phenotypes and can be used for rearing workers for behavioral studies. By regulating the quantity of food, *in vitro* reared larvae can develop into workers that are the same size as those reared in the hive.

Sugars played an important role in the development of ovaries. The higher the sugar content of the diet the more ovarioles developed. It seems that mixture of glucose and fructose and high sugar concentration had a positive effect on the ovariole numbers. The effects of nutrition on the development of ovaries and ovariole numbers are under investigation.

In summary, this technique is simpler than the previously reported techniques by feeding the larvae once a day, and does not require the use of fresh royal jelly. One or two day-old larvae should be grafted for rearing larvae *in vitro*. They should be kept at 34° C and 90% RH, and provisioned with larval diets. The larvae should be transferred to a pupation plate lined with filter paper when they begin defecating or deposit uric acid crystals. The filter paper should be changed the next day and pre-pupae should be transferred to new filter papers. They can pupate in Petri dishes as a group or individually in a 24 well plates. The Petri dishes and/or the 24 well plates should be inspected daily and the dead larvae or pupae should be removed. It should be kept in mind that the donor colonies should be free of any kind of diseases. Moreover, the grafting room, incubators, tubs, Petri plates, and grafting needles should also be clean, sterilized, and/or disinfected.
